# Association analysis of the *SLC22A11* (organic anion transporter 4) and *SLC22A12* (urate transporter 1) urate transporter locus with gout in New Zealand case-control sample sets reveals multiple ancestral-specific effects

**DOI:** 10.1186/ar4417

**Published:** 2013-12-23

**Authors:** Tanya J Flynn, Amanda Phipps-Green, Jade E Hollis-Moffatt, Marilyn E Merriman, Ruth Topless, Grant Montgomery, Brett Chapman, Lisa K Stamp, Nicola Dalbeth, Tony R Merriman

**Affiliations:** 1 Department of Biochemistry, University of Otago, 364 Leith St, North Dunedin 9016, New Zealand; 2 Queensland Institute of Medical Research, Brisbane, Queensland, Australia; 3 University of Otago, Christchurch, New Zealand; 4 Department of Medicine, University of Auckland, Auckland, New Zealand

## Abstract

**Introduction:**

There is inconsistent association between urate transporters *SLC22A11* (organic anion transporter 4 (OAT4)) and *SLC22A12* (urate transporter 1 (URAT1)) and risk of gout. New Zealand (NZ) Māori and Pacific Island people have higher serum urate and more severe gout than European people. The aim of this study was to test genetic variation across the *SLC22A11/SLC22A12* locus for association with risk of gout in NZ sample sets.

**Methods:**

A total of 12 single nucleotide polymorphism (SNP) variants in four haplotype blocks were genotyped using TaqMan® and Sequenom MassArray in 1003 gout cases and 1156 controls. All cases had gout according to the 1977 American Rheumatism Association criteria. Association analysis of single markers and haplotypes was performed using PLINK and Stata.

**Results:**

A haplotype block 1 SNP (*rs17299124*) (upstream of *SLC22A11*) was associated with gout in less admixed Polynesian sample sets, but not European Caucasian (odds ratio; OR = 3.38, *P* = 6.1 × 10^-4^; OR = 0.91, *P* = 0.40, respectively) sample sets. A protective block 1 haplotype caused the *rs17299124* association (OR = 0.28, *P* = 6.0 × 10^-4^). Within haplotype block 2 (*SLC22A11*) we could not replicate previous reports of association of *rs2078267* with gout in European Caucasian (OR = 0.98, *P* = 0.82) sample sets, however this SNP was associated with gout in Polynesian (OR = 1.51, *P* = 0.022) sample sets. Within haplotype block 3 (including *SLC22A12*) analysis of haplotypes revealed a haplotype with trans-ancestral protective effects (OR = 0.80, *P* = 0.004), and a second haplotype conferring protection in less admixed Polynesian sample sets (OR = 0.63, *P* = 0.028) but risk in European Caucasian samples (OR = 1.33, *P* = 0.039).

**Conclusions:**

Our analysis provides evidence for multiple ancestral-specific effects across the *SLC22A11/SLC22A12* locus that presumably influence the activity of OAT4 and URAT1 and risk of gout. Further fine mapping of the association signal is needed using trans-ancestral re-sequence data.

## Introduction

Hyperuricemia is a primary risk factor for gout [1], leading to the formation of monosodium urate crystals and gout in some people. Genome-wide association studies (GWAS) have identified 28 loci that account for approximately 6% of the variance in serum urate (SU) levels in European Caucasians [2]. Loci with the strongest effect encode proteins involved in secretion and renal filtration of uric acid. Two of these, *SLC2A9* and *ABCG2,* have a very strong effect in gout (risk allele odds ratio (OR) >2.0) in multiple ancestral groups [2-5], whilst a third locus *SLC17A1,* has a weaker effect (OR <1.5) [2,6,7]. The SLC22A11 (OAT4) and SLC22A12 (URAT1) genes are also associated with SU at a genome-wide level of significance [2]. However, evidence for association with gout is ambiguous.

SLC22A11 (OAT4) and SLC22A12 (URAT1) encode renal urate transporters located together on chromosome 11. SLC22A12 is expressed across multiple tissue types and developmental stages, whilst SLC22A11 expression is restricted to the kidneys and placenta [8]. URAT1 is a high-affinity apical urate transporter [9], whilst OAT4 transports multiple organic anions and has low affinity for urate [10]. Both URAT1 and OAT4 may play a role in gout secondary to diuretic use due to competitive transport of uric acid and diuretics [9,10].

Multiple single nucleotide polymorphisms (SNPs) at the SLC22A11/SLC22A12 locus have been associated with SU in European Caucasians, with two independent effects reported within the locus [2]. These SNPs are spread across a 1.3-Mb region and constitute four distinct linkage disequilibrium (LD) blocks, two of which contain *SLC22A11* and *SLC22A12*[2]. This suggests a complex pattern of association with SU in European Caucasians, at least. In non-European populations, studies specifically on the SLC22A12 gene have revealed associations with SU in Chinese, Korean and Japanese sample sets that are consistent with the those reported in European Caucasians (*rs505802* and SNPs in LD) [11-14] whilst additional associations are evident in Chinese (*rs475688* and *rs7932775*, in low LD with *rs505802*) [14,15].

Variants in the haplotype block that includes *SLC22A12* have previously been tested for association with gout in European Caucasian with no association of *rs505802* (OR = 0.99) [16] or *rs478607* (OR = 0.97) [2]. There is conflicting evidence for association of common *SLC22A12* variants with gout in Chinese sample sets [15,17]. Rare variants in the SLC22A12 locus have been associated with SU and gout in African-Americans [18] and gout in Japanese [19] and Micronesians [20]. At *SLC22A11*, Köttgen *et al.*[2] reported association of the minor (C) allele of *rs2078267* with prevalent gout in a meta-analysis of European Caucasian cases nested within population-based cohorts (OR = 1.16, *P* = 4.4 × 10^-6^). However, Stark *et al*. [16] reported no evidence for association of *rs17300741* (OR = 1.02, *P* = 0.80; in very strong LD with *rs2078267* (*r*^2^ = 0.96)) with gout. To our knowledge, no previous studies have tested for association of *SLC22A11* with SU and gout in non-European populations.

There is ambiguity regarding association of *SLC22A11* and *SLC22A12* with gout - previous reports have studied different population groups, with various methods for determination of gout; clinical ascertainment using 1977 American Rheumatism Association (ARA) criteria [15,17], self-report of physician-diagnosed gout, use of gout medications [2,18], a combination of self-report and examination of medical records [16]. Here our aim was to clarify the association with gout according to the ARA classification criteria at the SLC22A11-SLC22A12 locus in multiple ancestral groups drawn from the New Zealand (NZ) population, including Māori and Pacific Island. These groups have a prevalence of gout double that of European Caucasians [21], with earlier onset and more severe gout and a higher prevalence of co-morbidities [6].

## Methods

### Study participants

All gout cases (Additional file 1: Table S1; n = 1,003) had a confirmed diagnosis of gout as defined by the 1977 ARA preliminary classification criteria for acute gout [22] and were recruited from community-based settings, primary and secondary care. Controls (n = 1,156) self-reported having no history of arthritis and were convenience-sampled from workplaces and community focal points in the Auckland region of NZ. Co-morbid conditions (type 2 diabetes, renal disease, hypertension, dyslipidemia and heart disease) were self-reported. Ethical approval was given by the NZ Multi-Region Ethics Committee and all participants provided written informed consent for the collection of samples and subsequent analysis.

Participants were divided into four sample sets according to self-reported grandparental ancestry; NZ European Caucasian (420 cases, 638 controls), Eastern Polynesian (EP) - NZ Māori and Cook Island Māori (315 cases, 349 controls), Western Polynesian (WP) - Tongan, Samoan and Niuean (249 cases, 144 controls), and a small mixed eastern and western ancestry group (EP/WP) (19 cases, 25 controls) (Additional file 1: Table S1). These groupings were based on previous evidence for genetic heterogeneity between Eastern and Western Polynesia (see [5] and references therein). The EP sample set (predominantly NZ Māori) was further split into two groups according to Polynesian ancestry estimates obtained using 67 genomic control markers [6]. Individuals of higher EP ancestry formed the EP(High) sample set (256 cases, 187 controls) and those of lower ancestry formed the EP(Low) sample set (59 cases, 162 controls).

### SNP selection

Data on 1,000 genomes [23] for Caucasian (CEU) and Han Chinese (CHB), as the most closely related and available population to the NZ Māori and Pacific populations, was used to define haplotype blocks based on all the SNPs previously associated with SU in GWAS studies within the 1.3-Mb region of chromosome 11 (Additional file 2: Figure S1). Four distinct haplotype blocks were identified (block 1: approximately 26Kb upstream, Block 2: *SLC22A11,* Block 3: including *SLC22A12,* and block 4: approximately 840Kb downstream) (Additional file 1: Table S2) and this information was then used to select variants for genotyping (Additional file 2: Figure S2). SNPs were selected largely on the basis of previous literature reports of association with SU. For block 1 and block 4 both the SNP most associated with SU (*rs17299124;* β = −3.75 μmolL^-1^, *P* = 8.6 × 10^-16^ and *rs642803;* β = −2.56 μmolL^-1^, *P* = 4.5 × 10^-14^, respectively) and the most associated tag-SNP (in high LD with all other associated SNPs (*rs475414;* β = −2.38 μmolL^-1^, *P* = 3.1 × 10^-12^ and *rs12289836;* β = −2.62 μmolL^-1^, *P* = 6.6 × 10^-14^*,* respectively) were chosen [2,18,24]. For block 2 (*SLC22A11*) one tag-SNP common to CEU and CHB was identified (*rs693591*) and combined with the previously studied *rs17300741* and *rs2078267*[2,24]. One tag-SNP for block 3 (*SLC22A12*) was selected (*rs476037*) and included with the previously reported SNPs *rs3825018* (in complete LD with *rs505802*), *rs475688* and *rs7932775*[2,24]*.* A fifth SNP (*rs478607*) from block 3 was also added as representative of a second (weaker) effect potentially independent of *rs2078267* (*SLC22A12*) ([2]; β = −2.80 μmolL^-1^, *P* = 4.4 × 10^-11^) (Additional file 2: Figure S3, S4). Three of the twelve chosen SNPs have a predicted biological function according to FuncPred [25]; *rs3825018* is situated in a potential transcription factor binding site and may enhance or silence splicing, *rs7932775* may also enhance or silence splicing and *rs476037* is situated at a potential miRNA binding site.

### Genotyping

SNPs were genotyped using either TaqMan® assays (Applied Biosystems, Foster City, CA, USA) in a Lightcycler® 480 Real-Time PCR System (Roche, Indianapolis, IN, USA) (*rs693591* (assay ID C_925815_10), *rs17300741* (C_3234214_10), *rs3825018* (C_27162391_10)), *rs475688* (C_925797_10), *rs476037* (C_3108662_20), *rs7932775* (C_3108664_10), or the MassArray system (Sequenom iPLEXassay, San Diego, CA, USA): *rs17299124, rs642803, rs475414, rs12289836, rs478607* and *rs2078267*.

### Association analysis

Allelic ORs were obtained from a sex-adjusted logistic regression in PLINK [26]. These were relevant to the allele in-phase with the *rs3825018* G allele as determined using 1,000 genomes LD data. To assess experiment-wide significance a Bonferroni correction was applied to *P*-values. A conservative correction factor of 48 was applied to the single SNP analysis (*P* <0.00104; 12 SNPs meta-analyzed by inverse-variance weighting over four sample sets - EP(High), WP, EP/WP), all Polynesian (including EP(Low)), European Caucasian and EP(Low), and all five sample sets combined) and a factor of 12 for the haplotype analysis (*P* <0.0042; meta-analysis of three haplotype blocks over four sample sets). Interaction terms for co-morbid phenotypes and ancestry were included in the logistic regression model where appropriate.

Ancestrally defined sample sets were combined by an inverse-variance weighted fixed-effect method, using STATA 8.0 software [27]. A Q-statistic was calculated to determine the heterogeneity between cohorts and for SNPs showing heterogeneity (*P*_Het_ <0.05) the fixed-effect model was replaced with a random-effect model. Power was calculated for a nominal *P* = 0.05 (Additional file 2: Figure S5) [28]. All sample sets were adequately powered (>80%) to detect moderate effects (OR = 1.5) at minor allele frequency >0.1.

### Haplotype association analysis

Haplotype imputation by expectation maximization was performed in PLINK to produce most-likely haplotype calls (haplotypes with a frequency <0.01 were excluded). These data were then used to perform a sex-adjusted logistic regression in PLINK, producing ORs and *P-*values. Inverse-variance weighted fixed-effect analysis in STATA 8.0 was performed to combine the independent datasets (as described for the allelic association analysis).

### Locus-specific gout genetic risk score

A weighted genetic risk score was calculated using the most associated SNP in each of block one to three. The gout risk score calculation was ((*rs17299124*(C) × β) + (*rs2078267*(C) × β) + (*rs3825018*(G) × β)), where each SNP ID followed by an allele in brackets denotes the allele count per individual, and the β after each SNP denotes the natural log of the ancestry-specific OR for that allele. Individuals who did not have complete genotype information (9.22%) were excluded.

The mean genetic risk score was calculated for each sample set and a two-tailed *t*-test performed to identify any significant differences between case and control values. A further sex-adjusted logistic regression was performed to test association with gout. All risk-score analysis was conducted in STATA 8.0.

## Results

### Allelic associations

The European Caucasian sample set revealed nominally significant (*P* <0.05) associations with gout at two block-3 SNPs: *rs475688* and *rs7932775* (SLC22A12; OR = 1.26, *P* = 0.043; OR = 1.32, *P* = 0.033, respectively) (Table 1). The EP(High) sample set revealed four nominally significant associations within block 1 (*rs17299124;* OR = 2.77, *P =* 0.019), block 2 (SLC22A11) (*rs693591;* OR = 1.41, *P* = 0.031), and block 3 (SLC22A12) (*rs3825018;* OR = 1.46, *P* = 0.028, and *rs476037;* OR = 1.56, *P* = 0.022). The EP(Low) sample set showed no significant associations whereas the WP sample set revealed association with the block-1 SNP *rs17299124* (OR = 5.65, *P* = 0.011) and the block-3 SNP *rs7932775* (OR = 1.49, *P* = 0.019). The mixed Eastern and Western Polynesian sample set revealed a nominally significant association at *rs693591* (OR = 4.55, *P =* 0.025) (Table 1).

**Table 1:** Allelic association analysis of the twelve chromosome 11 SNPs with gout

				**East Polynesian (EP) (High)**				**East Polynesian (Low)**				**West Polynesian (WP)**				**East/West Polynesian**				**European Caucasian**			
	**SNP**^**b**^	**Chr:Pos**^**c**^	**Effect/Other**^**d**^	**Allele Freq.**		**OR**^**e **^**(95% CI)**	***P***^**e**^	**Allele Freq.**		**OR**^**e **^**(95% CI)**	***P***^**e**^	**Allele Freq.**		**OR**^**e **^**(95% CI)**	***P***^**e**^	**Allele Freq.**		**OR**^**e **^**(95% CI)**	***P***^**e**^	**Allele Freq.**		**OR**^**e **^**(95% CI)**	***P***^**e**^
				**Case**	**Con**			**Case**	**Con**			**Case**	**Con**			**Case**	**Con**			**Case**	**Con**		
1^a^			T/C	0.571	0.531	1.19	0.257	0.566	0.601	1.01	0.957	0.403	0.409	1.06	0.758	0.500	0.400	1.65	0.412	0.566	0.564	1.02	0.868
						(0.88,1.61)				(0.63,1.64)				(0.75,1.49)				(0.50,5.48)				(0.84,1.24)	
1	rs17299124	11:64256859	C/A	0.976	0.950	2.77	0.019	0.821	0.801	1.20	0.577	0.994	0.957	5.65	0.011	0.974	0.947	3.00	0.461	0.733	0.752	0.91	0.401
						(1.18,6.50)				(0.63,2.32)				(1.49,21.39)				(0.16,55.71)				(0.73,1.14)	
2	rs693591	11:64325069	C/G	0.522	0.449	1.41	0.031	0.315	0.291	1.13	0.630	0.373	0.367	1.00	0.992	0.526	0.260	4.55	0.025	0.139	0.135	0.95	0.733
						(1.03,1.92)				(0.68,1.89)				(0.72,1.40)				(1.21,17.06)				(0.72-1.26)	
2	rs17300741	11:64331462	A/G	0.819	0.789	1.30	0.187	0.604	0.557	1.29	0.312	0.808	0.830	0.76	0.213	0.806	0.750	1.42	0.595	0.476	0.439	1.05	0.611
						(0.88,1.91)				(0.78,2.14)				(0.49,1.17)				(0.39,5.24)				(0.87,1.27)	
2	rs2078267	11:64334114	C/T	0.938	0.910	1.78	0.063	0.651	0.606	1.19	0.526	0.980	0.944	2.20	0.071	0.921	0.886	1.21	0.815	0.479	0.456	0.98	0.816
						(0.97,3.26)				(0.70,2.00)				(0.94,5.17)				(0.24,6.11)				([0.81,1.18]	
3	rs3825018	11:64358809	G/A	0.739	0.674	1.46	0.028	0.500	0.447	1.37	0.206	0.726	0.712	1.16	0.404	0.842	0.660	1.83	0.316	0.305	0.267	1.22	0.065
						(1.04,2.06)]				(0.84,2.23)				(0.82,1.66)				(0.56,5.98)				(0.99,1.50)	
3	rs475688	11:64364291	T/C	0.433	0.427	1.10	0.543	0.370	0.295	1.66	0.073	0.409	0.461	0.78	0.145	0.556	0.420	1.72	0.391	0.269	0.236	1.26	0.043
						(0.80,1.52)				(0.95,2.89)				(0.56,1.09)				(0.50,5.97)				([1.01,1.57)	
3	rs7932775	11:64367862	C/T	0.496	0.478	1.03	0.866	0.373	0.320	1.36	0.278	0.489	0.415	1.49	0.019	0.500	0.396	1.00	1.000	0.213	0.174	1.32	0.033
						(0.76,1.40)				(0.78,2.35)				(1.07,2.09)				(0.35,2.90)				(1.02,1.69)	
3	rs476037	11:64369540	A/G	0.254	0.206	1.56	0.022	0.149	0.136	1.10	0.787	0.250	0.303	0.78	0.165	0.368	0.250	2.58	0.161	0.088	0.089	1.03	0.846
						(1.07,2.28)				(0.56,2.16)				(0.55,1.11)				(0.69,9.68)				(0.74,1.43)	
3	rs478607	11:64478063	G/A	0.241	0.268	0.85	0.359	0.279	0.184	1.72	0.080	0.196	0.166	1.48	0.085	0.211	0.225	0.82	0.766	0.167	0.144	1.27	0.077
						(0.60,1.20)				(0.94,3.15)				(0.95,2.31)				(0.23,2.98)				(0.98,1.66)	
4	rs12289836	11:65436888	A/G	0.621	0.622	0.94	0.711	0.660	0.631	1.07	0.810	0.693	0.746	0.80	0.237	0.790	0.725	1.22	0.766	0.645	0.683	0.85	0.109
						(0.68,1.30]				(0.62,1.85)				(0.55,1.16)]				(0.34,4.40)				(0.69,1.04)	
4	rs642803	11:65560620	T/C	0.454	0.458	1.00	0.975	0.500	0.474	1.26	0.364	0.413	0.421	0.92	0.624	0.500	0.368	0.71	0.620	0.448	0.474	0.93	0.467
						(0.73,1.36]				(0.77,2.07)				(0.66,1.29)				(0.18,2.75)				(0.77,1.13)	

^a^LD block as shown in Additional file 2: Figure S1 and S2, block 1: upstream, block 2: SLC22A11, block 3: SLC22A12, block 4: downstream. ^b^*rs2078267, rs3825018* and *rs476037* showed some evidence for a departure from Hardy-Weinberg equilibrium in the WP controls (*P* = 0.007, 0.001 and 0.006, respectively), *rs2078267* in the EP/WP cases (*P* = 0.005) and *rs476037* in the European Caucasian cases (*P =* 0.023). None were significant after correction for multiple testing (*P* <0.00042 (correction factor of 120; 10 datasets × 12 SNPs)). ^c^Base position is according to Genome Reference Consortium human genome build 37 (GRCh37). ^d^Effect/Other, alleles; all odds ratios (ORs) are calculated relevant to the effect allele defined as the allele in-phase with *rs3825018* G allele according to 1,000 Genomes linkage-disequilibrium data. ^e^OR, allelic OR (adjusted for sex); *P*, allelic asymptotic *P*-value. Genotyping success rate: rs475414: 97.55%; rs17299124: 94.35%; rs693591: 98.75%; rs17300741: 97.59%; rs2078267: 95.41%; rs3825018: 99.40%; rs475688: 98.75%; rs7932775: 92.40%; rs476037: 99.17%; rs478607: 98.01%; rs12289836: 97.82%; and rs642803: 97.73%. SNP, single nucleotide polymorphism; freq., frequency; Con, control.

Within block 1, observing that the effect of *rs1729914* was stronger in the less admixed Polynesian sample sets (EP(High), WP, EP/WP) (Table 1), we combined these sample sets by inverse-variance weighting for *rs17299124* and tested for association with gout in these sample sets, revealing experiment-wide evidence for association (Table 2; OR = 3.38, *P* = 6.1 × 10^-4^). The heterogeneity *P-*value of 0.010 (in the combined analysis of all ancestries) suggested an ancestral specific effect. Based on this, all SNPs were combined by inverse-variance weighting in four groups - less admixed Polynesian (EP(High), WP, EP/WP), all Polynesian (including EP(Low)), European Caucasian and EP(Low), and the five sample sets combined. Of the other SNPs, only block-3 *rs3825018* approached experiment-wide significance in all ancestries combined (OR = 1.27*, P* = 0.002). Because no nominal association with block 4 was seen in any sample set the relevant SNPs (*rs12289836* and *rs642803*) were not included in further analyses.

**Table 2:** Inverse-variance weighted analysis of allelic associations

				**European Caucasian and low ancestry Polynesian**^**d**^			**High ancestry Polynesian**^**e**^			**Polynesian**^**f**^			**All ancestries**^**g**^		
	**SNP**	**Chr:Pos**^**b**^	**Effect/Other**^**c**^	**OR**^**h**^	***P***^**h**^	**Het *P***^**h**^	**OR**^**h**^	***P***^**h**^	**Het *P***^**h**^	**OR**^**h**^	***P***^**h**^	**Het *P***^**h**^	**OR**^**h**^	***P***^**h**^	**Het *P***^**h**^
				**(95% CI**)			**(95% CI**)			**(95% CI**)			**(95% CI)**		
1^a^	rs475414	11:64241844	T/C	1.02	0.862	0.990	1.15	0.233	0.727	1.12	0.270	0.839	1.07	0.376	0.861
				(0.85,1.22)			(0.92,1.43)			(0.92,1.34)			**(**0.93,1.23)		
1	rs17299124	11:64256859	C/A	0.94	0.539	0.425	3.38	6.1 × 10^,4^	0.674	1.95	0.006	0.153	1.71	0.109	0.010
				(0.76,1.16)			(1.69,6.79)			(1.21,3.15)			**(**0.89,3.28)		
2	rs693591	11:64325069	C/G	0.99	0.942	0.559	1.25	0.051	0.053	1.23	0.047	0.113	1.12	0.167	0.087
				(0.78,1.26)			(1.00,1.57)			(1.00,1.51)			**(**0.95,1.33)		
2	rs17300741	11:64331462	A/G	1.08	0.402	0.446	1.04	0.793	0.173	1.10	0.469	0.254	1.07	0.396	0.388
				(0.90,1.29)			(0.78,1.38)			(0.86,1.40)			**(**0.92,1.24)		
2	rs2078267	11:64334114	C/T	1.00	0.999	0.499	1.84	0.012	0.804	1.51	0.022	0.591	1.08	0.370	0.169
				(0.84,1.20)			(1.14,2.95)			(1.06,2.14)			(0.91,1.28)		
3	rs3825018	11:64358809	G/A	1.24	0.028	0.660	1.33	0.020	0.568	1.34	0.008	0.767	1.27	0.002	0.822
				(1.02,1.50)			(1.05,1.69)			(1.08,1.66)			(1.10,1.48)		
3	rs475688	11:64364291	T/C	1.31	0.011	0.360	0.95	0.683	0.216	1.03	0.768	0.096	1.13	0.109	0.093
				(1.06,1.60)			(0.76,1.20)			(0.84,1.27)			(0.97,1.32)		
3	rs7932775	11:64367862	C/T	1.32	0.017	0.923	1.21	0.095	0.256	1.23	0.051	0.412	1.26	0.004	0.550
				(1.05,1.66)			(0.97,1.51)			(1.00,1.51)			(1.08,1.48)		
3	rs476037	11:64369540	A/G	1.05	0.770	0.874	1.25	0.480	0.014	1.19	0.461	0.036	1.08	0.423	0.070
				(0.78,1.40]			(0.67,2.34)			(0.75,1.89)			(0.89,1.31)		
3	rs478607	11:64478063	G/A	1.33	0.020	0.371	1.04	0.785	0.148	1.13	0.337	0.109	1.19	0.056	0.167
				(1.05,1.70)			(0.79,1.36)			(0.88,1.44)			(1.00,1.43)		
4	rs12289836	11:65436888	A/G	0.87	0.156	0.435	0.89	0.327	0.717	0.91	0.423	0.791	0.88	0.085	0.863
				(0.72,1.05)			(0.70,1.13)			(0.73,1.14)			(0.76,1.02)		
4	rs642803	11:65560620	T/C	0.97	0.728	0.267	0.95	0.662	0.862	1.00	0.982	0.726	0.96	0.586	0.818
				(0.81,1.16)			(0.76,1.19)			(0.81,1.23)			(0.84,1.11)		

^a^LD block as shown in Additional file 2: Figure S1 and S2, block 1: upstream, block 2: SLC22A11, block 3: SLC22A12, block 4: downstream. ^b^Base position is according to Genome Reference Consortium human genome build 37 (GRCh37). ^c^Effect/Other, effect allele and other allele, all odds ratios (ORs) are calculated relevant to the effect allele defined as the allele in-phase with *rs3825018* G allele according to 1,000 genomes linkage disequilibrium data. ^d^Combining EP(L) and NZ European Caucasian sample sets. ^e^Combining the EP(H), WP and EP/WP sample sets. ^f^Combining the Eastern Polynesian EP high (EP(H)), EP low (EP(L)), Western Polynesian (WP) and EP/WP sample sets. ^g^Combining the EP(H), EP(L), WP, EP/WP and NZ European Caucasian sample sets. ^h^OR: inverse-variance weighted analysis OR (adjusted for sex); *P*: inverse-variance weighted analysis *P*-value; Het *P*: heterogeneity *P*-value, inverse-variance weighted analyses were reanalyzed using a random-effects model if the Het *P* was <0.05.

Block-1 to −3 SNPs *rs17299124, rs2078267* and *rs3825018* were tested for association with SU levels in controls (Additional file 1: Table S3). The only nominally significant associations observed (*P* <0.05) were with *rs2078267* in less admixed Polynesian and total Polynesian (β = 0.044 mmolL^-1^, *P* = 0.007 and β = 0.030 mmolL^-1^, *P* = 0.022 respectively). The minor allele increased SU, consistent with the increased risk mediated for gout by the minor allele in these sample sets (Table 2). These associations were, however, not significant after correcting *P-*values for multiple testing (n = 12).

Interaction terms were included in the sex-adjusted logistic regression analyses in order to test for interaction between genotype (of the most associated SNP in blocks 1 to 3), gout and body mass index, type 2 diabetes, dyslipidemia, heart disease and hypertension in the European Caucasian, less admixed Polynesian and total Polynesian samples (Additional file 1: Table S4). There were no significant interactions observed (after correcting for the number of tests done, n = 45). We also tested for interaction with ancestry with significant interaction observed, after correcting for the number of tests done (n = 3), with ancestry for block-1 SNP *rs17299124* (*P*_c_ = 9.9 × 10^-6^) and block-2 SNP *rs2078267* (*P*_c_ = 2.6 × 10^-5^). The interaction with ancestry at *rs17299124* was consistent with the *P*_Het_ = 0.01 in the inverse-variance weighted analysis with the *rs2078267* association also specific to Polynesian (Table 2). The *P*_Interaction_ at *rs3825018* was 0.033.

### Haplotype association analysis

We initially combined by inverse-variance weighting haplotypes in the same combinations of sample sets as used in Tables 2 and 3. At block 1 there was a haplotype (*rs475414* (allele T) - *rs17299124* (allele A)) that exhibited significant heterogeneity (*P*_Het_ = 0.007) in the combined analysis of all ancestries. Consistent with the *rs17299124* data the effect, which was protective, was restricted to the less admixed Polynesian sample sets (OR = 0.28, *P* = 6.0 × 10^-4^). This protective T-A haplotype was rarer in the less admixed Polynesian sample set (Table 4 control frequency 0.044-0.052) compared with EP(Low) 0.203 and European Caucasian 0.245.

**Table 3:** Inverse-variance weighted analysis of block- 1 to -3 haplotypes

	**European Caucasian and low ancestry Polynesian**^**b**^			**High ancestry Polynesian**^**c**^			**Polynesian**^**d**^			**All ancestries**^**e**^		
**Hap**^***a***^	**OR**^**f**^	**P**^**f**^	**Het P**^**f**^	**OR**^**f**^	**P**^**f**^	**Het P**^**f**^	**OR**^**f**^	**P**^**f**^	**Het P**^**f**^	**OR**^**f**^	**P**^**f**^	**Het P**^**f**^
	**(95% CI)**			**(95% CI)**			**(95% CI)**			**(95% CI)**		
Block 1 (*rs475414*|*rs17299124*)												
C|C	0.94	0.501	0.795	0.89	0.305	0.884	0.91	0.349	0.936	0.92	0.242	0.978
	(0.78,1.13)			(0.71,1.11)			(0.74,1.11)			(0.80,1.06)		
T|A	1.07	0.549	0.379	0.28	6.0 × 10^,4^	0.512	0.50	0.006	0.120	0.56	0.103	0.007
	(0.86,1.32)			(0.14,0.58)			(0.31,0.82)			(0.28,1.12)		
T|C	1.02	0.848	0.584	1.30	0.022	0.914	1.29	0.019	0.965	1.14	0.093	0.572
	(0.83,1.25)			(1.04,1.63)			(1.04,1.56)			(0.98,1.33)		
Block 2 (*rs693591*|*rs17300741*|*rs2078267*)												
C|A|C	0.97	0.800	0.726	1.24	0.070	0.128	1.20	0.083	0.219	1.10	0.253	0.179
	(0.76,1.24)			(0.98,1.56)			(0.98,1.49)			(0.93,1.30)		
G|A|C	1.08	0.468	0.842	0.78	0.037	0.446	0.83	0.083	0.368	0.94	0.440	0.204
	(0.88,1.31)			(0.61,0.99)			(0.67,1.03)			(0.81,1.10)		
G|G|C	,	,	,	1.35	0.093	0.369	1.31	0.113	0.509	,	,	,
				(0.95,1.91)			(0.94,1.83)					
G|G|T	0.97	0.706	0.682	0.57	0.022	0.797	0.69	0.042	0.600	0.91	0.252	0.316
	(0.81,1.15)			(0.35,0.92)			(0.49,0.99)			(0.77,1.07)		
Block 3 (*rs3825018*|*rs475688*|*rs7932775*|*rs476037*|*rs478607*)												
A|C|T|G|A	0.82	0.040	0.953	0.76	0.035	0.272	0.77	0.024	0.451	0.80	0.004	0.594
	(0.67,0.99)			(0.59,0.98)			(0.62,0.97)			(0.68,0.93)		
G|C|C|G|A	1.06	0.816	0.425	1.31^g^	0.105	0.969	1.23^g^	0.183	0.543	1.22^h^	0.142	0.746
	(0.66,1.69)			(0.95,1.81)			(0.91,1.67)			(0.94,1.60)		
G|C|C|G|G	,	,	,	1.33	0.116	0.702	1.29	0.151	0.752	,	,	,
				(0.93,1.90)			(0.91,1.81)					
G|T|C|G|A	,	,	,	0.93	0.730	0.905	,	,	,	,	,	,
				(0.63,1.39)								
G|T|C|G|G	1.41	0.007	0.248	0.63	0.028	0.821	0.86	0.690	0.028	1.01	0.984	0.014
	(1.10,1.82)			(0.41,0.95)			(0.41,1.80)			(0.61,1.66)		
G|T|T|A|A	1.01	0.947	0.731	1.19	0.573	0.029	1.07	0.590	0.062	1.06	0.579	0.118
										(0.87,1.29)		
	(0.75,1.36)			(0.66,2.14)			(0.84,1.36)					

^a^Block 1 (*rs475414*|*rs17299124*), block 2 (*rs693591*|*rs17300741*|*rs2078267*), block 3. (*rs3825018*|*rs475688*|*rs7932775*|*rs476037*|*rs478607*) excluding frequency <0.01. ^b^Combining the Eastern Polynesian (EP) low (EP(L)) and NZ European Caucasian sample sets. ^c^Combining the EP high (EP(H)), Western Polynesian (WP) and EP/WP sample sets. ^d^Combining the EP(H), EP(L), WP, and EP/WP sample sets. ^e^Combining the EP(H), EP(L), WP, EP/WP and NZ European Caucasian sample sets. ^f^OR: Inverse-variance weighted analysis odds ratio (adjusted for sex); *P*: inverse-variance weighted analysis *P*-value; Het *P*: heterogeneity *P*-value, inverse-variance weighted analyses were reanalyzed using a random-effects model if the Het *P* was <0.05. ^g^EP/WP cohort was excluded from this meta-analysis owing to no female controls having the G|C|C|G|A haplotype.

**Table 4:** Sample set-specific haplotype association analysis at haplotype blocks 1 to 3

	**East Polynesian (High)**^**b**^				**East Polynesian (Low)**^**c**^				**West Polynesian**				**East/West Polynesian**				**European Caucasian**			
**Hap**^***a***^	**Freq.**		**OR**^**d **^**(95% CI)**	***P***^**d**^	**Freq.**		**OR**^**d **^**(95% CI)**	***P***^**d**^	**Freq.**		**OR**^**d **^**(95% CI)**	***P***^**d**^	**Freq.**		**OR**^**d **^**(95% CI)**	***P***^**d**^	**Freq.**		**OR**^**d **^**(95% CI)**	***P***^**d**^
	**Case**	**Con**			**Case**	**Con**			**Case**	**Con**			**Case**	**Con**			**Case**	**Con**		
Block 1 (*rs475414*|*rs17299124*)																				
C|C	0.437	0.472	0.86	0.321	0.434	0.397	1.00	0.988	0.598	0.595	0.94	0.745	0.500	0.579	0.75	0.648	0.430	0.450	0.93	0.471
			(0.63,1.16)				(0.61,1.62)				(0.67,1.34)				(0.22,2.60)				(0.76,1.14)	
T|A	0.024	0.051	0.35	0.017	0.179	0.203	0.81	0.525	0.004	0.044	0.12	0.009	0.026	0.053	0.33	0.461	0.262	0.245	1.10	0.393
			(0.15,0.83)				(0.42,1.56)				(0.03,0.59)				(0.02,6.19)				(0.88,1.38)	
			1.34	0.063	0.387	0.400	1.18	0.562	0.396	0.361	1.24	0.228	0.474	0.368	1.53	0.486	0.302	0.301	1.00	0.984
T|C	0.539	0.477	(0.98,1.82)				(0.67,2.09)				(0.88,1.76)				(0.46,5.08)				(0.80,1.24)	
Block 2 (*rs693591*|*rs17300741*|*rs2078267*)																				
C|A|C	0.525	0.458	1.38	0.047	0.315	0.298	1.05	0.849	0.373	0.371	1.02	0.933	0.528	0.283	3.49	0.063	0.139	0.134	0.95	0.698
			(1.00,1.90)				(0.63,1.77)				(0.72,1.43)				(0.93,13.01)				(0.72,1.25)	
G|A|C	0.299	0.350	0.77	0.136	0.287	0.272	1.13	0.654	0.436	0.461	0.83	0.288	0.278	0.478	0.38	0.106	0.329	0.302	1.07	0.543
			(0.54,1.09)				(0.66,1.95)				(0.60,1.17)				(0.12,1.23)				(0.87,1.32)	
G|G|C	0.11	0.102	1.09	0.734	0.046	0.040	0.94	0.914	0.17	0.121	1.72	0.032	0.111	0.13	0.76	0.758	,	,	,	,
			(0.66,1.83)				(0.28,3.14)				(1.05,2.83)				(0.14,4.30)					
G|G|T	0.061	0.090	0.56	0.070	0.352	0.381	0.87	0.607	0.019	0.043	0.50	0.117	0.083	0.109	0.93	0.932	0.525	0.556	0.98	0.830
			(0.30,1.05)				(0.52,1.47)				(0.21,1.19)				(0.19,4.68)				(0.81,1.18)	
Block 3 (*rs3825018*|*rs475688*|*rs7932775*|*rs476037*|*rs478607*)																				
A|C|T|G|A	0.273	0.327	0.71	0.053	0.510	0.545	0.81	0.420	0.290	0.303	0.89	0.520	0.167	0.421	0.29	0.088	0.678	0.717	0.82	0.059
			(0.50,1.01)				(0.48,1.36)				(0.61,1.28)				(0.07,1.21)				(0.67,1.01)	
G|C|C|G|A	0.179	0.136	1.26	0.283	0.078	0.115	0.78	0.582	0.159	0.129	1.37	0.214	0.139	0.053	,	0.997	0.035	0.024	1.19	0.534
			(0.83,1.93)				(0.32,1.88)				(0.83,2.26)								(0.68,2.08)	
G|C|C|G|G	0.116	0.108	1.14	0.599	0.039	0.038	0.82	0.767	0.142	0.114	1.54	0.100	0.111	0.105	1.71	0.659	,	,	,	,
			(0.69,1.89)				(0.23,2.98)				(0.92,2.59)				(0.16,18.73)					
G|T|C|G|A	0.057	0.068	0.84	0.574	0.000	0.016	,	0.998	0.111	0.102	1.01	0.971	0.139	0.053	0.96	0.968	,	,	,	,
			(0.46,1.55)								(0.59,1.73)				(0.14,6.67)					
G|T|C|G|G	0.098	0.139	0.64	0.068	0.226	0.131	2.03	0.036	0.026	0.034	0.63	0.354	0.056	0.105	0.31	0.301	0.161	0.133	1.33	0.039
			(0.40,1.03)				(1.05,3.95)				(0.24,1.68)				(0.04,2.83)				(1.01,1.75)	
G|T|T|A|A	0.266	0.213	1.53	0.030	0.128	0.131	0.90	0.776	0.264	0.314	0.78	0.183	0.361	0.263	2.28	0.300	0.088	0.087	1.04	0.836
			(1.04,2.24)				(0.44,1.85)				(0.55,1.12)				(0.48,10.80)				(0.74,1.44)	

^a^Hap, haplotypes listed in alphabetical order; those with a frequency <0.01 were excluded. ^b^East Polynesian (High), individuals with a STRUCTURE ancestry level ≥0.67. ^c^East Polynesian (Low), individuals with a STRUCTURE ancestry level <0.67. ^d^OR, odds ratio (adjusted by sex); *P*: asymptomatic *P*-value. freq., frequency; Con, control.

At block 2 no haplotype association was observed. At block 3 the only other experiment- wide significant association was observed, a protective haplotype in the combined sample set (A-C-T-G-A; OR = 0.80, *P* = 0.004). This haplotype was more common in European Caucasian (control frequency of 0.717) than Polynesian (control frequency range of 0.303-0.545). The block-3 G-T-C-G-G haplotype showed evidence for heterogeneity (*P*_Het_ = 0.014) in the combined analysis of all ancestries – closer examination showed that this owed to a risk effect in European Caucasian and more admixed Polynesian (EP(Low)) (OR = 1.41, *P* = 0.007) and a protective effect in less admixed Polynesian (OR = 0.63, *P* = 0.028) (Tables 3 and 4). The frequency of this haplotype varied considerably, even within the Polynesian sample sets (Table 4 control frequency: WP 0.034, EP(High) 0.139, EP(Low) 0.131, European Caucasian 0.133).

### Gout genetic risk-score association

A weighted genetic risk score was calculated to determine the cumulative effect of the approximately 360-Kb (block 1 to block 3) region on gout risk, using the most associated marker in each ancestral block. Genetic risk scores ranged from 0 to 18.03 with no European Caucasian individual risk score being >7 (range 0 to 6.21). In each sample set the average case risk-score was significantly larger than the average control risk-score (Table 5). There was a significant association between gout and the genetic risk score in EP(High) (OR = 1.22, *P* = 0.002) and WP (OR = 1.14, *P* = 0.041) but not in European Caucasian (OR = 1.01, *P* = 0.80), EP(Low) (OR = 1.03, *P* = 0.77) or the small EP/WP sample set (OR = 1.32, *P* = 0.21).

**Table 5:** Genetic risk score analysis

	**Mean ± standard deviation**		**ū**_**1**_-**ū**_**2**_^**a**^	***t***^**a**^	***P***^**a**^	**OR**^**b**^	***P***^**b**^	**Variance explained (%)**^**c**^
	**Case**	**Control**	**(95% CI)**^**a**^			**(95% CI)**		
East Polynesian (H)	10.93 ± 1.56	10.45 ± 1.91	0.48	2.71	0.007	1.22	0.002	1.38
			(0.13, 0.83)			(1.07-1.39)		
East Polynesian (L)	4.81 ± 1.70	4.68 ± 1.96	0.13	0.47	0.64	1.03	0.77	0.09
			(-0.44, 0.71)			(0.84-1.26)		
West Polynesian	17.24 ± 1.21	16.57 ± 2.95	0.67	2.43	0.016	1.14	0.041	1.89
			(0.13, 1.22)			(1.01-1.30)		
Polynesian	11.16 ± 1.54	10.23 ± 2.04	0.93	1.54	0.14	1.32	0.21	5.11
			(-0.31, 2.18)			(0.85-2.05)		
European Caucasian	3.02 ± 1.60	2.94 ± 1.52	0.08	0.75	0.45	1.01	0.80	0.04
			(-0.13, 0.28)			(0.93-1.11)		

^a^ū_1_-ū_2_, difference in means (case (ū1) minus control (ū2)); *t*, *t*-value, *P*, two-tailed *t*-test *P*-value. ^b^OR, odds ratio (adjusted by sex); *P*, logistic regression *P*-value. ^c^Based on pseudo-*R*^2^ calculated during logistic regression.

## Discussion

We analyzed three haplotype blocks - a region upstream of *SLC22A11* containing no known genes (block 1), *SLC22A11* (block 2) and *SLC22A12* (block 3). Collectively our data indicate a complex pattern of association that is ancestry-dependent, with risk appearing to be driven by protective haplotypes that are at a higher frequency in European Caucasian than in people of Polynesian ancestry. The genetic risk-score analysis (Table 5) emphasized the relative important of the locus on the risk of gout in less admixed Polynesian (% variance explained 1.4 to 1.9) compared to the more admixed Polynesian and European Caucasian sample sets (<0.1% variance explained). Common variation in this locus is likely to be part of the explanation for why Polynesian people exhibit higher SU and a higher prevalence of gout [6,21].

The block-1 region has previously never been studied for association with gout. The single SNP data of *rs17299124* showed the strongest effect in the less admixed Polynesian sample sets (OR = 3.38) with non-significant effects closer to OR = 1.00 in the more admixed Eastern Polynesian and European Caucasian sample sets (OR = 1.20 and 0.91, respectively). This SNP is therefore a novel candidate risk variant to test for association with gout in South East Asian populations (ancestrally related to Polynesian). Haplotype analysis indicated that this effect in less admixed Polynesians is driven by a genetic variant that protects from gout. The lack of any known gene within the region (Ensembl release 70, January 2013) suggests that the effect seen at *rs17299124* may operate by influencing the expression of a nearby gene, perhaps *SLC22A11*. Previously *rs2186571* has been associated with SU levels in the Pacific Micronesian population of Kosrae [20]. This SNP, which maps several hundred kilobases upstream of *SLC22A11*, further implicates this region in the etiology of gout. There are two inversions and a deletion spanning this region [29], possibly able to cause a difference in the regulation of *SLC22A11* or *SLC22A12* expression.

At block 2 (*SLC22A11*) *rs2078267* has previously been convincingly associated with gout in European Caucasian (OR = 0.88, *P* = 2.3 × 10^-5^; [2]) but the NZ European Caucasian sample set is inconsistent with these findings (OR = 0.98, *P* = 0.82). While low power may be a factor, we expected to observe an effect size consistent with that reported by Köttgen *et al.*[2] (OR = 1.14 to the C allele). It is possible that differences in phenotype among gout cases are important at *SLC22A11.* In the Köttgen *et al*. study the association was restricted to the prevalent gout cases (OR = 0.86, *P* = 4.4 × 10^-6^) and not observed in the incident cases (OR = 0.96, *P* = 0.55). Ascertainment in the prevalent group was dominated by a combination of self-report or use of urate-lowering medication and colchicine (prescribed for alleviating pain from acute gout), whereas the incident group was ascertained, as were the NZ gout cases (OR = 0.98, *P* = 0.82), by use of the ARA classification criteria for gout, a superior ascertainment method [30] (although the NZ cases were ascertained by clinical examination whereas the incident ones by a self-administered questionnaire [31]). It is therefore counter-intuitive that the association of *SLC22A11* should be restricted to gout cases ascertained by less stringent criteria. Understanding this intriguing paradox may reveal new information on the role of *SLC22A11* (*OAT4*) in the control of SU and in the risk of gout. There are physiological data showing that OAT4 mediates hydrochlorothiazide-induced hyperuricemia [10] and also evidence for interaction of *rs2078267* with diuretic use in determining the risk of gout [32]. It is possible that non-additive interaction with diuretics could be a factor in the inconsistencies. In the Polynesian analysis there was nominal evidence for association with gout at *rs2078267*, with the minor allele conferring risk (OR = 1.51, *P* = 0.022), consistent with the urate-increasing effect of this allele in European Caucasians [2]. However this association was not significant when corrected for multiple testing. Given the lower allele frequency of this variant in Polynesians (<0.10 in the less admixed samples), a larger sample size is required for more robust conclusions about the possible role of *SLC22A11* in gout in Polynesian people.

At *SLC22A12* the clearest findings came from analysis of haplotype association. There was a gout-protective variant associated in the combined analysis (A-C-T-G-A; OR = 0.80, *P* = 0.004), with a consistent direction of effect in Polynesians and European Caucasians. There was also a haplotype with ancestral specific effects; G-T-C-G-G was protective in the less admixed Polynesian sample sets, yet conferred risk in the more admixed EP and European Caucasian sample sets. Our data therefore suggest that multiple common variants within the SLC22A12 locus contribute to the risk of gout, in a population-dependent manner. In the European Caucasian sample set there were nominal *P-*values for *rs475688* and *rs7932775* of 0.043 (OR = 1.26) and 0.033 (OR = 1.32), respectively. This is, to our knowledge, the first report of nominally significant association of *SLC22A12* with gout in European Caucasians. However, given the likely weak effect, confirmation of this will require larger gout sample sets.

The block-3 SNP *rs475688*, in moderate LD (*r*^2^ = 0.80) with *rs3825018* in European Caucasians but low LD in CHB and Polynesians (*r*^2^ = 0.33 and 0.36, respectively) (Additional file 2: Figures S2 and S3), showed weak evidence for association with gout in the NZ European Caucasian sample set (OR = 1.26, *P* = 0.04) consistent with the minor (T) allele increasing SU levels in Caucasians (β = 3.28 μmolL^-1^, *P* = 1.3 × 10^-17^) [2]. This variant, however, has been associated with gout and SU in Han Chinese and Solomon Island (Melanesian) sample sets with the T allele conferring protection against gout (OR = 0.54, *P* = 1 × 10^-4^) and decreasing serum urate levels (β = −11.90 μmolL^-1^, *P* = 0.02) [15], a direction of association opposite to that observed in NZ European Caucasians. This opposite effect between Polynesians and European Caucasians at *rs475688* is consistent with the opposing direction of association seen with the block-3 G-T-C-G-G haplotype (*rs475688* is the second marker) between less admixed Polynesians, and European Caucasians and the more admixed Eastern Polynesians. It will be informative to test this haplotype for association with SU and gout in Asian and other Pacific populations.

## Conclusions

We present several important findings: 1) novel association of *rs17299124* upstream of *SLC22A11* with gout in Polynesians and not Caucasians, which is driven by a protective effect; 2) evidence for association of the *SLC22A11* SNP *rs2078267* with gout in Polynesians with the previously reported association in European Caucasians not replicated; and 3) the *SLC22A12* analysis was the first report of nominally significant association with gout in European Caucasians, with the haplotype analysis suggesting multiple alleles conferring risk at *SLC22A12* in an ancestry-specific manner.

## Abbreviations

ARA: American Rheumatism Association; CEU: Centre d’Etude du Polymorphisme Humain from Utah; CHB: Han Chinese from Beijing; EP: Eastern Polynesian; GWAS: Genome-wide association studies; LD: Linkage disequilibrium; NZ: New Zealand; OAT4: Organic anion transporter 4; OR: Odds ratio; SNP: Single nucleotide polymorphism; SU: Serum urate; URAT1: Urate transporter 1; WP: Western Polynesian; NZ: New Zealand.

## Competing interests

The authors declare that they have no competing interests.

## Authors’ contributions

TJF, JEH-M, and TRM helped to design the study, oversee its execution, and prepare the manuscript. AP-G, MEM, RT, LKS and ND helped to provide clinical recruitment and prepare the manuscript. BC and GWM helped to collect data and prepare the manuscript. All authors read and approved the final manuscript.

## Supplementary Material

Additional file 1: Table S1 Demographic and clinical characteristics of study participants. **Table S2:** Haplotype block summary. **Table S3:** Association of block-1 to −3 single nucleotide polymorphisms (SNPs) with serum urate in control individuals. **Table S4:** Interaction analysis between genotype, gout and co-morbid phenotypes. Click here for file

Additional file 2: Figure S1 Intermarker linkage disequilibrium of single nucleotide polymorphisms (SNPs) previously associated with serum urate. **Figure S2:** Common linkage disequilbrium block haplotypes in European Caucasian and Han Chinese. **Figure S3:** Intermarker linkage disequilibrium between the 12 genoytped SNPs in European Caucasian and Han Chinese. **Figure S4:** Intermarker linkage disequilibrium between the 12 genoytped SNPs in New Zealand sample sets. **Figure S5:** Power calculations. Click here for file
