# Supplementary material for: Association analysis of the SLC22A11 (organic anion transporter 4) and SLC22A12 (urate transporter 1) urate transporter locus with gout in New Zealand case-control sample sets reveals multiple ancestral-specific effects
Source: Arthritis Res Ther. 2013 Dec 23;15(6):R220. doi: 10.1186/ar4417 (PMC3978909; doi:10.1186/ar4417)
Supplement: Additional file 1: Table S1 — Demographic and clinical characteristics of study participants. Table S2: Haplotype block summary. Table S3: Association of block-1 to −3 single nucleotide polymorphisms (SNPs) with serum urate in control individuals. Table S4: Interaction analysis between genotype, gout and co-morbid phenotypes. [file ar4417-S1.docx]

**Table S1**: Demographic and clinical characteristics

|  |  | East Polynesian | |  | West Polynesian | |  | East/West Polynesian | |  | European Caucasian | |
| --- | --- | --- | --- | --- | --- | --- | --- | --- | --- | --- | --- | --- |
|  |  | Case | Control |  | Case | Control |  | Case | Control |  | Case | Control |
| Total Participants |  | 315 | 349 |  | 249 | 144 |  | 19 | 25 |  | 420 | 638 |
| BASELINE INFORMATION |  |  |  |  |  |  |  |  |  |  |  |  |
| Age (years), mean ± standard deviation^a^ |  | 40 ± 14.971  (75.24) | 42 ± 13.935  (95.99) |  | 35 ± 11.652  (99.20) | 39 ± 12.689  (96.53) |  | 28 ± 7.775  (100.00) | 31 ± 12.784  (96.00) |  | 47 ± 15.897  (99.76) | 45 ± 17.465  (93.57) |
| Gender (% Males) |  | 76.41  (95.56) | 33.53  (98.28) |  | 93.57  (100.00) | 56.12  (96.53) |  | 100.00  (100.00) | 52.00  (100.00) |  | 83.49  (99.52) | 42.10  (97.18) |
| BMI^b^ (kg/m^2^), mean ± standard deviation |  | 34.90 ± 7.460  (78.73) | 32.29 ± 7.800  (55.01) |  | 37.31 ± 8.630  (97.59) | 34.28 ± 6.659  (72.92) |  | 37.60 ± 9.220  (100.00) | 34.29 ± 5.314  (60.00) |  | 30.35 ± 5.177  (99.29) | 27.71 ± 5.685  (20.38) |
| Stated Ancestry (%)^c^, mean ± standard deviation |  | 0.79 ± 0.259  (80.63) | 0.62 ± 0.304  (97.99) |  | 0.93 ± 0.148  (100.00) | 0.88 ± 0.210  (99.31) |  | 0.89 ± 0.178  (100.00) | 0.72 ± 0.268  (100.00) |  | 0.97 ± 0.123  (100.00) | 0.98 ± 0.089  (63.01) |
| Serum Urate Level at Collection (mmolL^-1^), mean ± standard deviation |  | 0.42 ± 0.107  (41.90) | 0.34 ± 0.087  (38.97) |  | 0.45 ± 0.111  (65.46) | 0.37 ± 0.070  (64.58) |  | 0.51 ± 0.075  (52.63) | 0.41 ± 0.117  (52.00) |  | 0.39 ± 0.104  (43.10) | 0.29 ± 0.077  (19.75) |
| % First Degree Relative(s) with Gout |  | 63.64  (76.83) | 34.02  (55.59) |  | 53.31  (97.19) | 23.76  (70.14) |  | 68.42  (100.00) | 23.08  (52.00) |  | 42.51  (96.90) | 13.64  (20.69) |
| COMORBIDITIES |  |  |  |  |  |  |  |  |  |  |  |  |
| % Type 2 Diabetes |  | 28.00  (79.37) | 9.20  (74.79) |  | 15.57  (97.99) | 12.20  (85.42) |  | 26.32  (100.00) | 4.35  (92.00) |  | 14.83  (99.52) | 3.75  (91.85) |
| % Dyslipidemia |  | 51.81  (79.05) | 12.16  (73.07) |  | 52.74  (95.18) | 12.90  (86.11) |  | 55.56  (94.74) | 8.70  (92.00) |  | 47.58  (98.57) | 14.34  (88.56) |
| % Heart Problems |  | 35.94  (81.27) | 3.14  (73.07) |  | 15.16  (97.99) | 1.59  (87.50) |  | 16.67  (94.74) | 0.00  (88.00) |  | 35.89  (99.52) | 5.89  (98.43) |
| % Taking Diuretics^d^ |  | 27.31  (68.57) | 3.89  (51.58) |  | 10.28  (85.94) | 3.92  (70.83) |  | 22.22  (94.74) | 0.00  (52.00) |  | 23.56  (86.90) | 3.20  (19.59) |
| % High Blood Pressure |  | 61.11  (80.00) | 16.54  (72.78) |  | 44.26  (97.99) | 18.25  (87.50) |  | 47.37  (100.00) | 12.5  (96.00) |  | 47.61  (99.52) | 16.94  (85.11) |
| % Kidney Problems |  | 26.59  (80.00) | 1.58  (54.44) |  | 16.74  (95.98) | 0.95  (72.92) |  | 27.78  (94.74) | 0.00  (52.00) |  | 19.57  (98.57) | 2.29  (20.53) |
| GOUT CHARACTERISTICS |  |  |  |  |  |  |  |  |  |  |  |  |
| Serum Urate Level at Diagnosis (mmolL^-1^), mean ± standard deviation |  | 0.53 ± 0.129  (55.56) | - |  | 0.56 ± 0.104  (75.10) | - |  | 0.63 ± 0.148  (63.16) | - |  | 0.48 ± 0.123  (63.57) | - |
| Number of Attacks Per Year, mean ± standard deviation |  | 10 ± 26.727  (73.65) | - |  | 13 ± 33.802  (98.39) | - |  | 8 ± 7.981  (94.74) | - |  | 6 ± 20.961  (97.38) | - |
| % Tophus Present at Recruitment |  | 43.28  (75.56) | - |  | 51.81  (100.00) | - |  | 31.58  (100.00) | - |  | 36.67  (100.00) | - |
| % On Urate Lowering Drugs^d^ |  | 75.29  (80.95) | - |  | 81.05  (99.60) | - |  | 73.68  (100.00) | - |  | 74.88  (99.52) | - |

In brackets after each summary statistic is the percentage (%) of participants for whom data were available.

^a^Cases: age at first attack; Controls: age at collection.

^b^BMI: body mass index.

^c^Based on self-reported ancestry of grandparents.

^d^Question asks “Have you ever taken…” some individuals may not have been taking the specified medication at time of collection.

**Table S2**: Haplotype block summary

|  |  | Start^a^ | End^a^ |  | Length (Mb) |  | Genes^b^ |
| --- | --- | --- | --- | --- | --- | --- | --- |
| Block 1 |  | 64223527 | 64273034 |  | 49.51 |  | - |
| Block 2 |  | 64299330 | 64334114 |  | 34.78 |  | SLC22A11 |
| Block 3 |  | 64351721 | 64581658 |  | 229.94 |  | SLC22A12, NRXN2, RASGRP2, PYGM, SF1, MAP4K2, MEN1 |
| Block 4 |  | 65419364 | 65566719 |  | 147.36 |  | RELA, KAT5, RNASEH2C, PP1030, OVOL1 |

^a^ Start and end positions are based on most upstream and downstream SNPs included in the block, base position is according to Genome Reference Consortium human genome build 37 (GRCh37).

^b^ According to Ensembl and HapMap gene position data.

**Table S3:** Association of block 1-3 SNPs with serum urate levels (mmolL^-1^) in control individuals

|  |  |  |  |  | Block 1: rs17299124 | | |  | Block 2: rs2078267 | | |  | Block 3: rs3825018 | | |
| --- | --- | --- | --- | --- | --- | --- | --- | --- | --- | --- | --- | --- | --- | --- | --- |
|  |  | Mean urate (mmolL^-1^) ± SD | n |  | β (mmolL^-1^) | *P* | Het-*P*^1^ |  | β (mmolL^-1^) | *P* | Het-*P*^1^ |  | β (mmolL^-1^) | *P* | Het-*P*^1^ |
| European Caucasian |  | 0.289 ± 0.077 | 126 |  | -0.010 [-0.028; 0.008] | 0.279 | - |  | 0.008 [-0.008; 0.024] | 0.327 | - |  | 0.006 [-0.011; 0.024] | 0.483 | - |
| European Caucasian and Low Ancestry Polynesian |  | 0.292 ± 0.080 | 160 |  | -0.010 [-0.026; 0.007] | 0.262 | 0.899 |  | 0.008 [-0.007; 0.023] | 0.318 | 0.886 |  | 0.004 [-0.012; 0.019] | 0.653 | 0.498 |
| High Ancestry Polynesian |  | 0.362 ± 0.081 | 208 |  | 0.029 [-0.007; 0.065] | 0.112 | 0.975 |  | 0.044 [0.012; 0.076] | 0.007 | 0.931 |  | 0.007 [-0.006; 0.021] | 0.292 | 0.054 |
| Polynesian |  | 0.354 ± 0.085 | 242 |  | 0.015 [-0.013; 0.043] | 0.292 | 0.671 |  | 0.030 [0.004; 0.055] | 0.022 | 0.527 |  | 0.005 [-0.007; 0.018] | 0.402 | 0.094 |

1 Heterogeneity *P* after combination of individual sample sets by inverse-variance weighted analysis.

**Table S4**: *P*_Interaction_ between genotype, gout and various co-morbid phenotypes

|  | *rs17299124* | | | | | *rs2078267* | | | | | *rs3825018* | | | | |
| --- | --- | --- | --- | --- | --- | --- | --- | --- | --- | --- | --- | --- | --- | --- | --- |
|  | BMI | T2D | Hypertension | Dyslipidemia | Heart | BMI | T2D | Hypertension | Dyslipidemia | Heart | BMI | T2D | Hypertension | Dyslipidemia | Heart |
| European | 0.90 | 0.30 | 0.22 | 0.50 | 0.73 | 0.34 | 0.53 | 0.91 | 0.85 | 0.12 | 0.65 | 0.95 | 0.74 | 1.00 | 0.10 |
| Polynesian (High) | 0.92 | 0.98 | 0.18 | 0.17 | 0.11 | 0.15 | 0.51 | 0.52 | 0.21 | 0.03 | 0.51 | 0.009 | 0.18 | 0.61 | 0.76 |
| Polynesian | 0.80 | 0.56 | 0.56 | 0.63 | 0.26 | 0.47 | 0.86 | 0.30 | 0.48 | 0.47 | 0.92 | 0.08 | 0.20 | 0.66 | 0.98 |
