# Supplementary material for: Association analysis of the SLC22A11 (organic anion transporter 4) and SLC22A12 (urate transporter 1) urate transporter locus with gout in New Zealand case-control sample sets reveals multiple ancestral-specific effects
Source: Arthritis Res Ther. 2013 Dec 23;15(6):R220. doi: 10.1186/ar4417 (PMC3978909; doi:10.1186/ar4417)
Supplement: Additional file 2: Figure S1 — Intermarker linkage disequilibrium of single nucleotide polymorphisms (SNPs) previously associated with serum urate. Figure S2: Common linkage disequilbrium block haplotypes in European Caucasian and Han Chinese. Figure S3: Intermarker linkage disequilibrium between the 12 genoytped SNPs in European Caucasian and Han Chinese. Figure S4: Intermarker linkage disequilibrium between the 12 genoytped SNPs in New Zealand sample sets. Figure S5: Power calculations. [file ar4417-S2.docx]

**Supplemental Figure 1**: Inter-marker linkage disequilibrium of all chromosome 11 SNPs previously associated with serum urate levels.

Figures generated using 1000 Genomes genotype data in Haploview 4.2, approximate gene positions are marked (SLC22A11: left, SLC22A12: right) along with defined LD Blocks (Block 1 (49.5kb): left, Block 2 (34.8kb): middle left, Block 3 (229.9kb): middle right), Block 4 (147.4kb): right).

^a^European Caucasian: Utah residents (CEU) with Northern and Western European ancestry.

^b^Chinese: Han Chinese in Beijing, China.

**Supplemental Figure 2:** Linkage disequilibrium block haplotypes with a frequency >10% in either European Caucasian (CEU) or Han Chinese (HCB) individuals.

The Figure was generated using 1000 Genomes data and Haploview 4.2 with the SNPs genotyped in this study highlighted. The alleles of each SNP are arbitrarily represented by either blue or red and the frequency of each haplotype shown for European Caucasian (left) / Chinese (right). In block 1 all major haplotypes of both ancestral groups are identified by the combination of the SNPs used; in blocks 2 and 3 this is achieved only in Chinese; in block 4 not all haplotypes can be distinguished in either population by the genotyped SNPs.

**Supplemental Figure 3:** Inter-marker linkage disequilibrium between the 12 genotyped SNPs.

Figures generated using 1000 Genomes genotype data in Haploview 4.2, approximate gene positions are marked (SLC22A11: left, SLC22A12: right)

^a^European Caucasian: Utah residents (CEPH) with Northern and Western European ancestry.

^b^Chinese: Han Chinese in Beijing, China.

**Supplemental Figure 4**: Inter-marker linkage disequilibrium between the 12 genotyped SNPs in New Zealand sample sets.

Figures generated using Haploview 4.2, approximate gene positions are marked (SLC22A11: left, SLC22A12: right).

^a^European Caucasian: New Zealand Caucasian cases and controls

^b^Polynesian: All Polynesian cases and controls (combined)

**Supplemental Figure 5**: Power across a range of minor allele frequencies for weak (OR=1.2), moderate (OR=1.5), and strong (OR=2.0) effect sizes.

^a^European Caucasian sample set power calculations, based on 420 cases and 638 controls.

^b^Combined Polynesian sample set power calculations, based on 583 cases and 518 controls.

**Supplemental Figure 1**


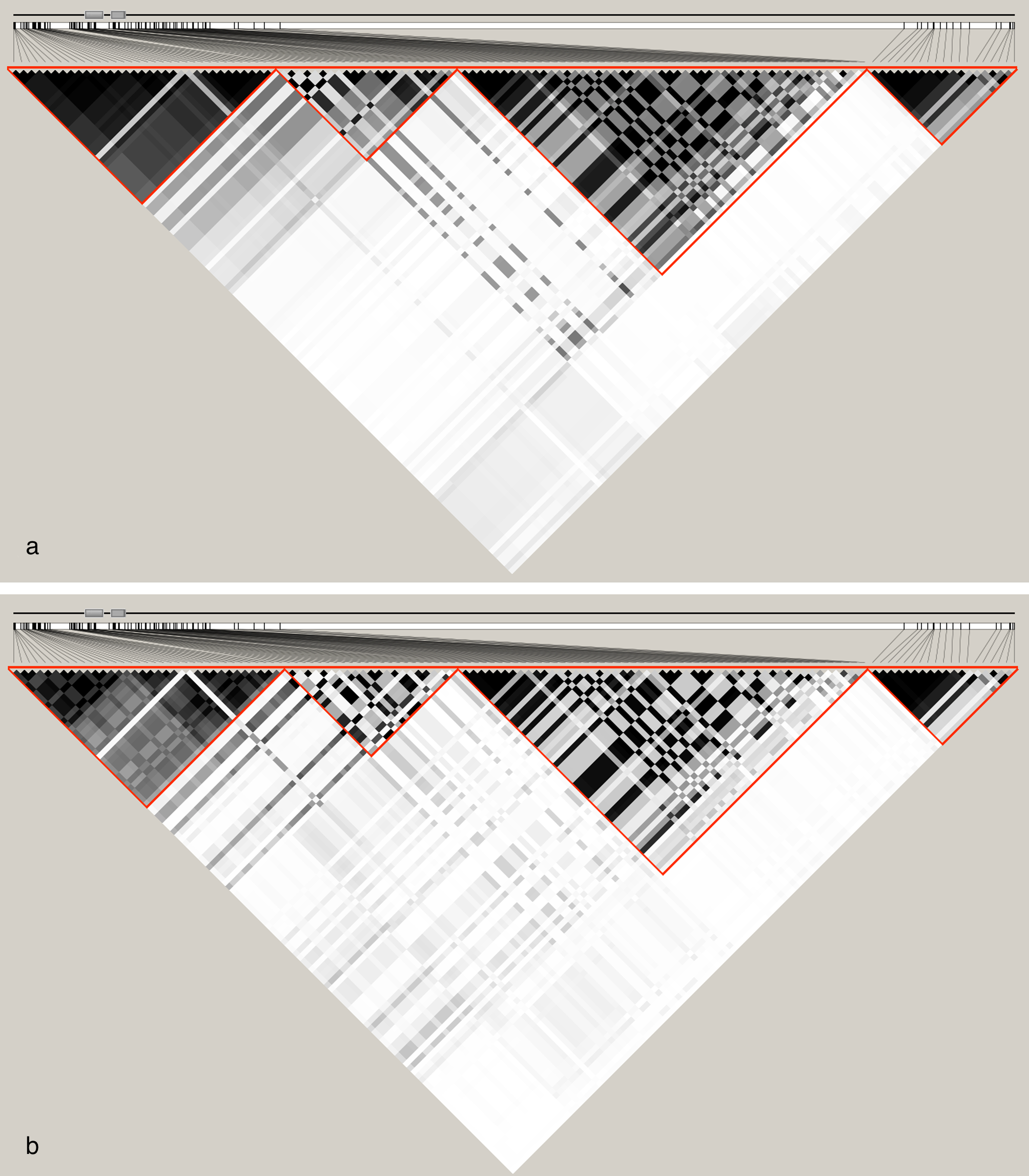


**Supplemental Figure 2**

**
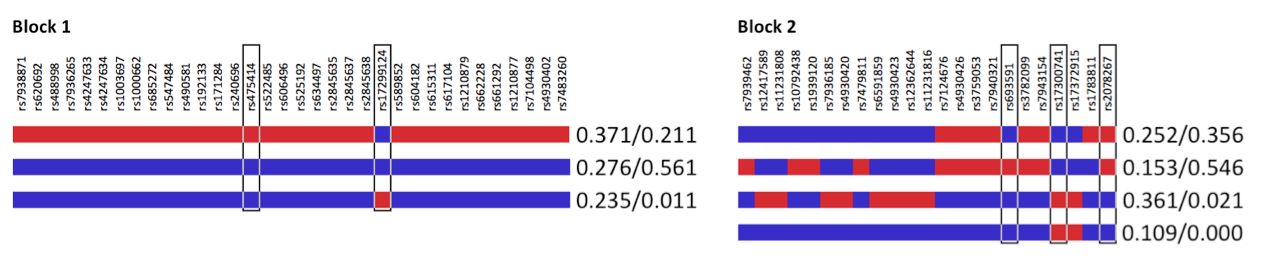
**

**
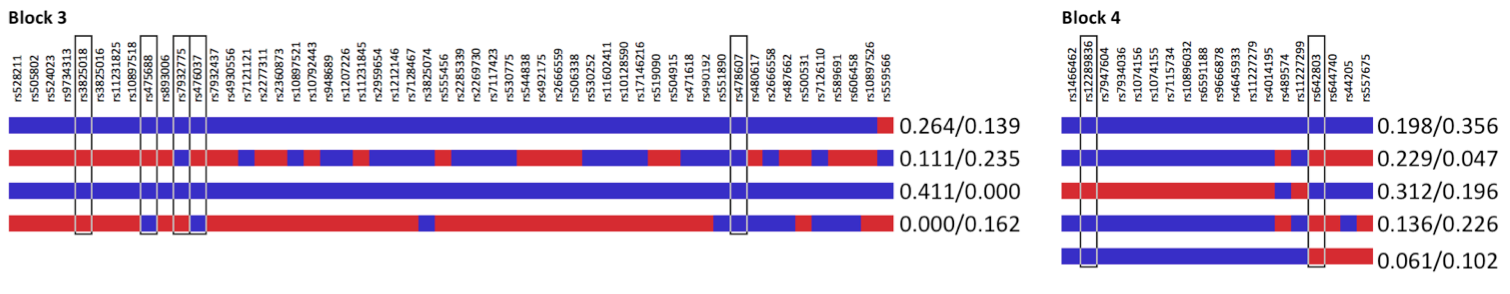
**

**Supplemental Figure 3**


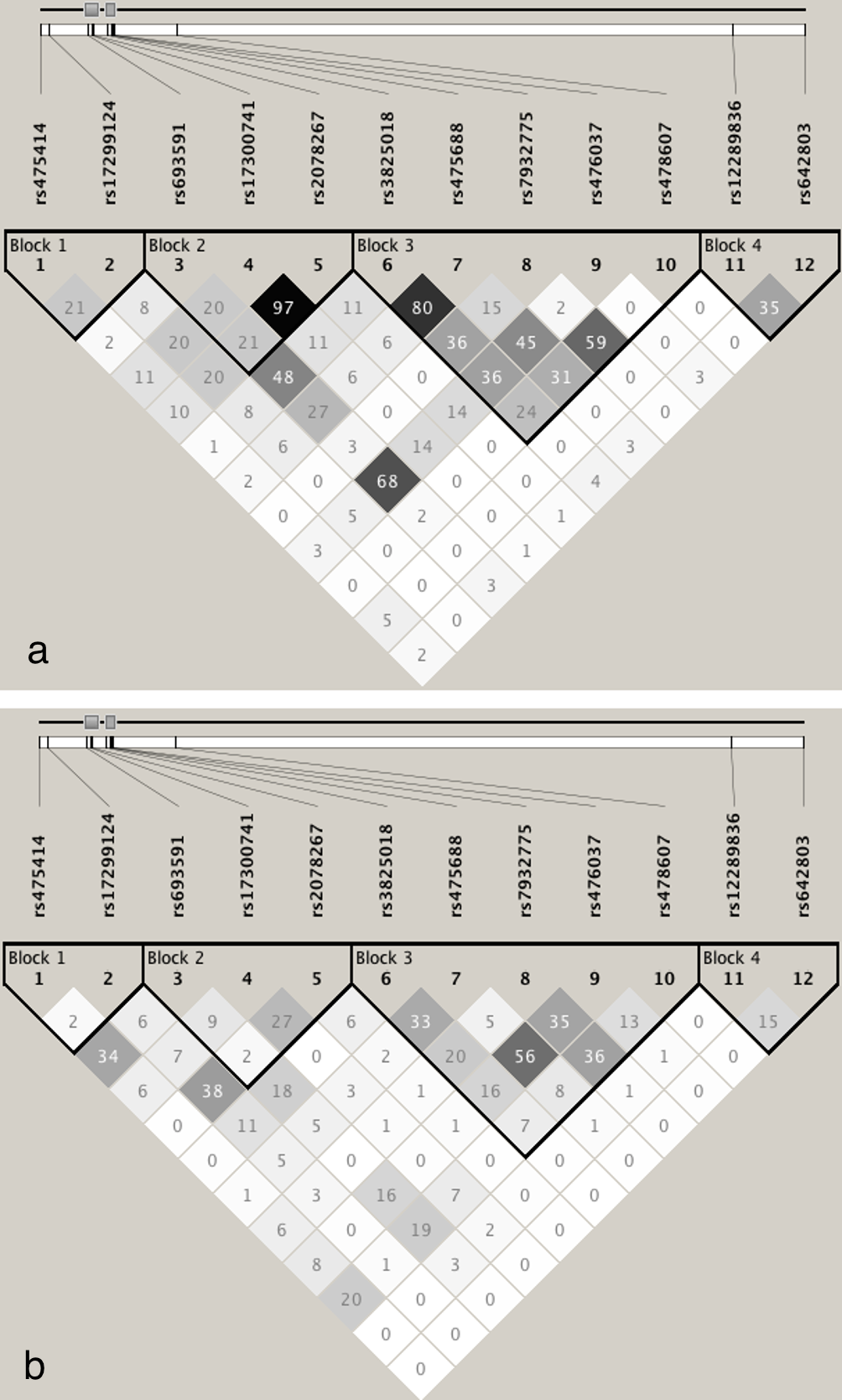


**Supplemental Figure 4**


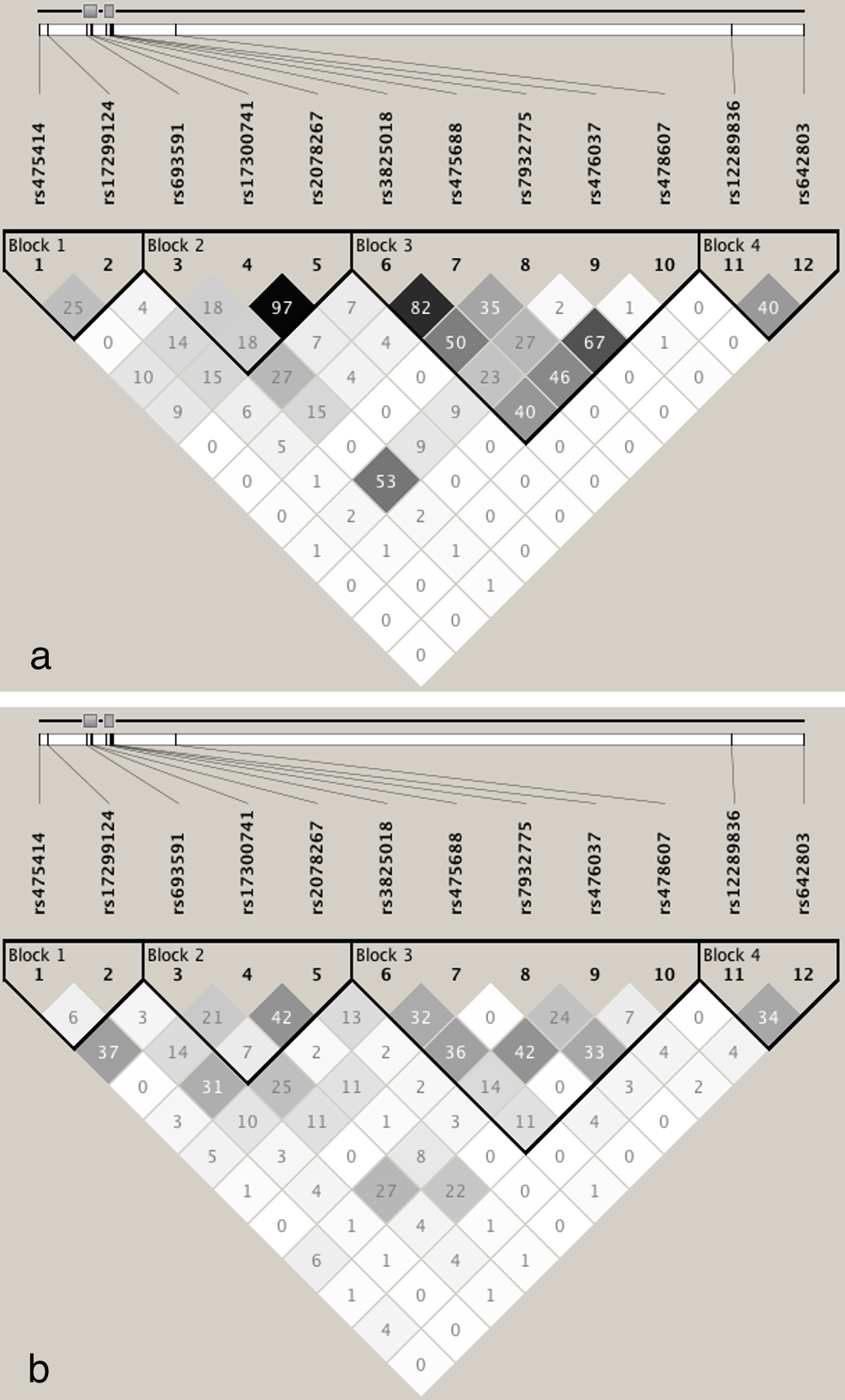


**Supplemental Figure 5**

**
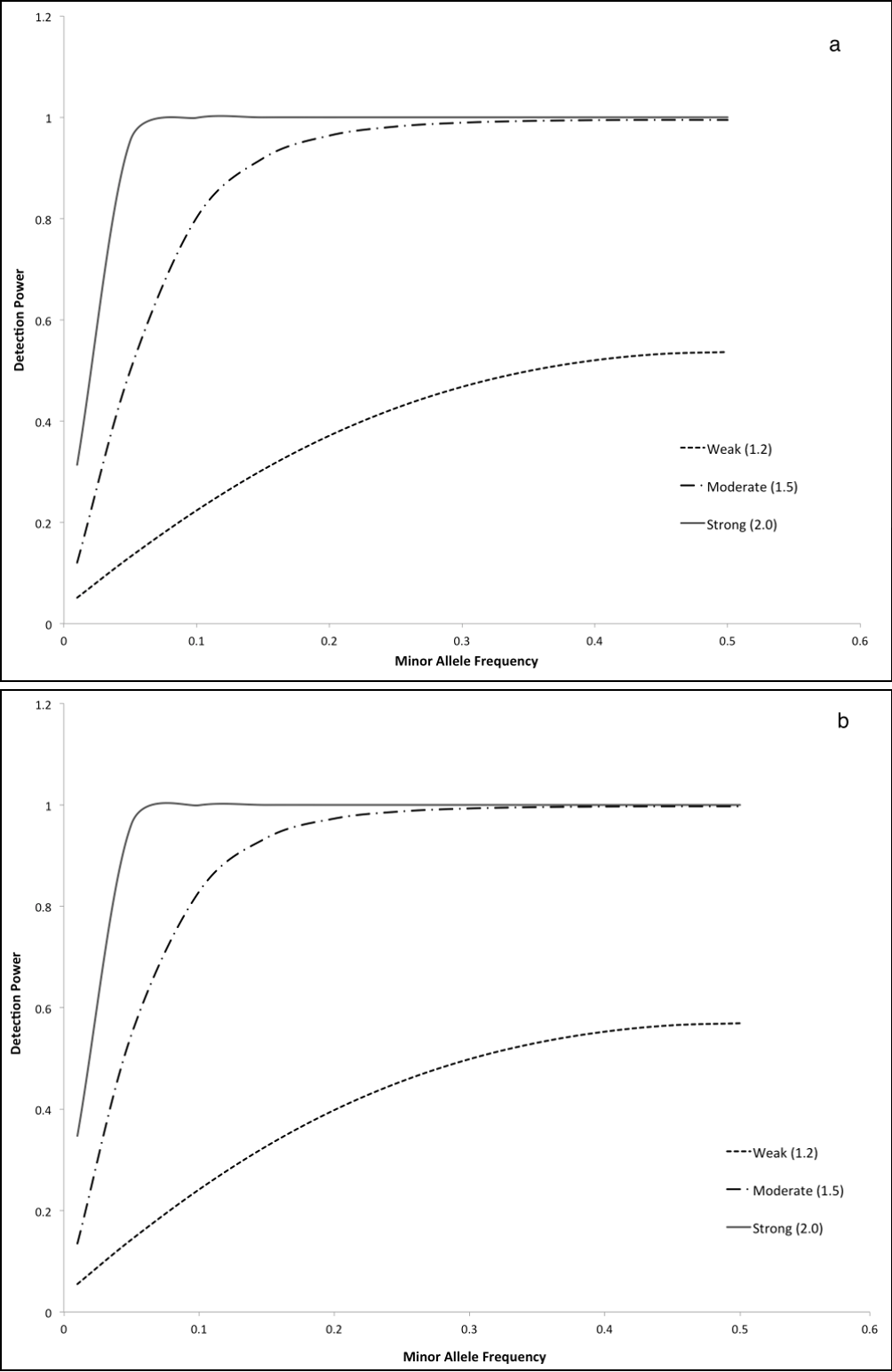
**
